# Nanostructured materials characterized by scanning photoelectron spectromicroscopy

**DOI:** 10.3762/bjnano.16.54

**Published:** 2025-05-23

**Authors:** Matteo Amati, Alexey S Shkvarin, Alexander I Merentsov, Alexander N Titov, María Taeño, David Maestre, Sarah R McKibbin, Zygmunt Milosz, Ana Cremades, Rainer Timm, Luca Gregoratti

**Affiliations:** 1 Elettra – Sincrotrone Trieste S.C.p.A., SS14-Km163.5 in Area Science Park, 34149 Trieste, Italy; 2 M.N. Miheev Institute of Metal Physics of Ural Branch of Russian Academy of Sciences, 620990, Ekaterinburg, Russia; 3 Department of Materials Physics, Universidad Complutense de Madrid, Plaza Ciencias 1, 28040 Madrid, Spain; 4 Center for Cooperative Research on Alternative Energies (CIC energiGUNE), Basque Research and Technology Alliance (BRTA), Alava Technology Park, Albert Einstein 48, 01510 Vitoria-Gasteiz, Spain; 5 Department of Physics, Lund University, 221 00 Lund, Sweden

**Keywords:** nanostructured materials, operando, oxides, scanning photoelectron spectromicroscopy, semiconductor nanowires, transition metal dichalcogenides, XPS

## Abstract

Nanostructured materials play a key role in modern technologies adding new functionalities and improving the performance of current and future applications. Due to their nature resulting in diffused heterogeneous structures (chemical and electronic composition typically organized in phases or building blocks) characterizing these materials needs state of the art technologies which combine nanometer spatial resolution, environmental reliability, and operando capabilities. Scanning photoelectron spectromicroscopy (SPEM) is one of the characterization tools that combine high spectral resolution X-ray photoelectron spectroscopy with submicron spatial resolution. In particular, the SPEM equipment hosted at the ESCA microscopy beamline at Elettra is capable of in situ and operando analysis regardless of sample morphology. The review presents three different case studies illustrating the capabilities of SPEM in the investigation of catalytic materials in different conditions and processes.

## Introduction

Nanometer or micrometer-sized materials play a key role in modern technologies in the search of new routes for unforeseen performances generating breakthroughs in societal challenges [1]. When composed of different elements, molecules, or compounds, these materials often show a regular and/or diffused heterogeneous structure based on elementary building blocks (e.g., crystallites or atomic/molecular groups) forming the entire solid or, in other cases, just their surfaces. The characterization of the building blocks is of paramount importance to deeply understand their functionalities and mutual interactions when they are part of a nanostructured body. The building blocks may differ in their atomic structure, crystallographic orientation, chemical composition, and charge distribution, to list the most important features. If the building blocks are crystallites, any change in the structure or chemical composition may lead to the formation of incoherent or coherent interfaces among them which may influence the final properties of the material. Often the volumes of the materials formed by similar building blocks, then having similar properties, are called phases from which another possible definition of these heterogeneous materials such as multi-phase materials comes [2–4].

Nanostructured materials are the playground for the advancement of some key technologies associated with electronics, energy conversion and storage, and many other fields due to their unique physical properties which may open unforeseen doors for technical and performance advances [5–8]. The full chain of steps necessary for the implementation of nanostructured materials in devices, which include the synthesis, characterization, and processing, are part of an emerging and rapidly growing field referred to as nanotechnology. However, despite the large efforts of the scientific and industrial communities in the development of new nanostructured materials and related technologies, the main obstacle in the application still resides in having a full control of their parameters in the production step. This is important to guarantee the availability of bulk materials with reproducible and fully reliable engineered properties and technological functionalities.

In the last decades, many characterization tools used to investigate chemical, electronic, or structural properties of materials have been developed or upgraded to match the requirements imposed by nanotechnology. Imaging capabilities covering from meso- to atomic scales, spatially resolved spectroscopies with enhanced sensitivities are examples of capabilities that modern techniques of characterization in nanotechnology must possess. X-ray photoelectron spectroscopy (XPS) is still one of the fundamental tools for chemical and electronic characterization of surfaces and subsurface layers. In the last three to four decades, several important upgrades have spread the original ability of XPS of chemical analysis to include, for instance, band mapping through angle resolved measurements (ARPES), spin detection, and imaging or spectromicroscopy at a nanoscale spatial resolution [9–10]. It is worth noting that several improvements have been developed at synchrotron light facilities where unique properties of X-ray radiation can be found. Scanning photoelectron microscopy (SPEM) combines XPS analysis with lateral resolution; chemical imaging as well as XPS spectroscopy at nanoscale sized areas can be performed providing fine chemical and electronic analysis of samples regardless of their morphology, which often limits the capabilities of other microscopy techniques [11]. This work reports three examples of SPEM experiments focused on the characterization of nanostructured materials. Measurements were performed at the SPEM microscope hosted at the ESCA microscopy beamline at the Elettra synchrotron research center. In the first example, chemical heterogeneous layered transition metal dichalcogenides were analyzed, showing the importance of spatial resolution in their characterization. In the second example, an operando chemical and electronic characterization of an InP nanowire heterostructure device under applied bias is reported. In the last example, the results of a SPEM investigation of self-organized NiO microcavity arrays exhibiting dimensions in the micrometric range concludes this overview of nanostructured materials characterized by SPEM.

## Results and Discussion

### Chemical heterogeneities in layered transition metal dichalcogenides

**Figure 1:**
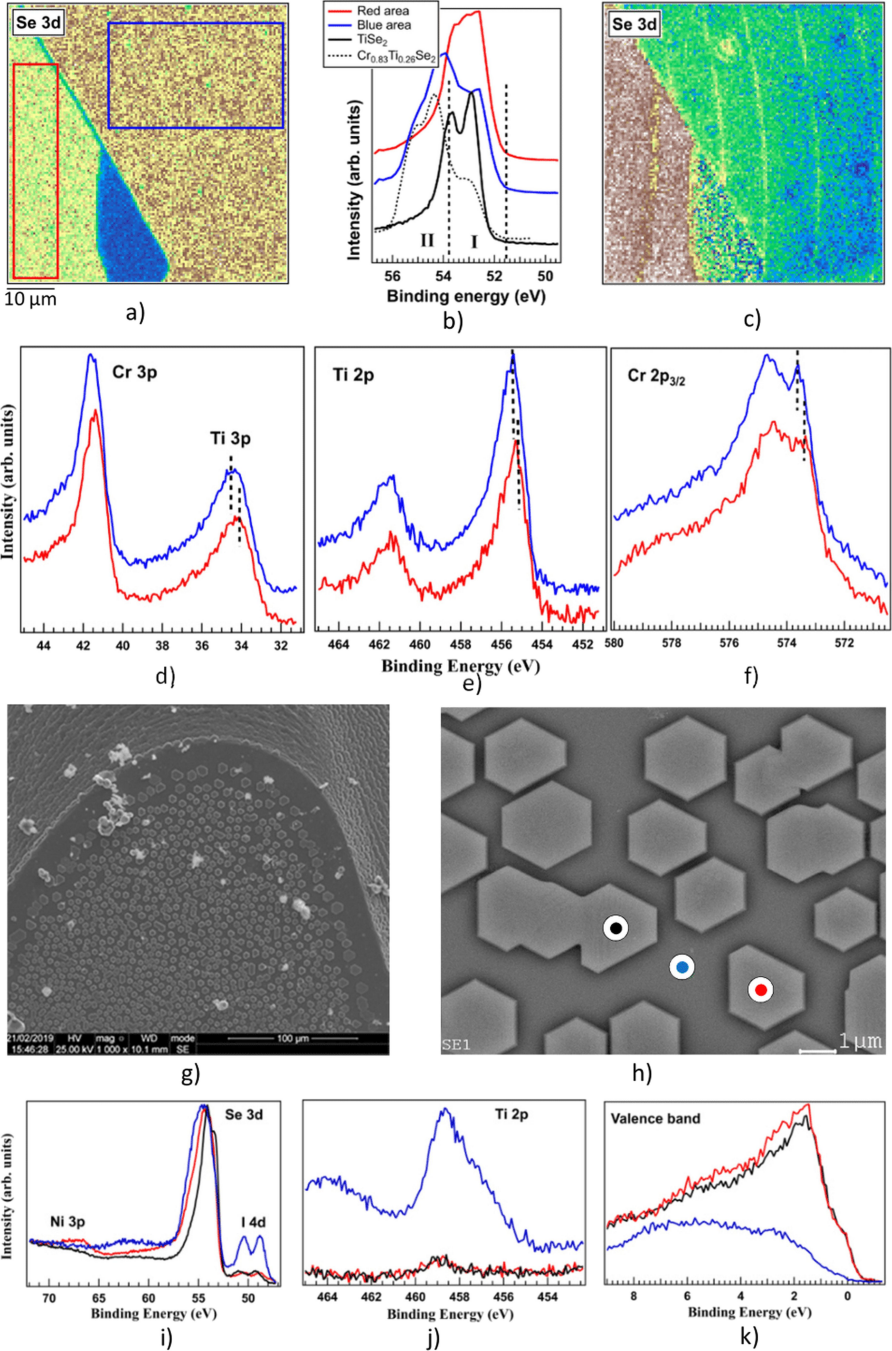
a) SPEM image of the Cr_0.78_Ti_0.36_Se_2_ single crystal obtained at the area corresponding to the Se 3d core-level spectrum (Se 3d contrast). b) Se 3d core level spectra for Cr_0.78_Ti_0.36_Se_2_ collected from areas marked as coloured rectangles in panel a) along with Se 3d spectra for previously investigated Cr_0.83_Ti_0.26_Se_2_ (black dashed line) and TiSe_2_ (black line) [12]; energy regions I and II correspond to different Se 3d_5/2_ states. c) Map in the Se 3d contrast plotted as II/III intensity ratio in each point, where II and III are the intensities of the Se 3d_5/2_ peak in the energy regions I and II (panel b)), respectively. Ti 3p and Cr 3p (d), Ti 2p (e) and Cr 2p_3/2_ (f) core-level spectra for Cr_0.78_Ti_0.36_Se_2_ collected from the “red” and “blue” regions (panel a)). SEM images (g), h)) of the as-grown (Fe,Ni)_0.25_TiSe_2_ single crystal. Se 3d and Ni 3p (i)), Ti 2p (j)) core level and valence band (k)) spectra for (Fe,Ni)_0.25_TiSe_2_ in red, blue and black points (panel h)). Figure 1a–f were reprinted from [13], *J. Phys. Chem. Solids*, vol. 160, by A. I. Merentsov; A. S. Shkvarin; M. S. Postnikov; L. Gregoratti; M. Amati; P. Zeller; P. Moras; A. N. Titov, “Studying the heterogeneity of the Cr_x_Ti_1-x_Ch_2_ (Ch = S, Se) single crystals using X-ray scanning photoemission microscopy“, article no. 110309, Copyright (2022), with permission from Elsevier. This content is not subject to CC BY 4.0. Figure 1g–k were reprinted from [14], *Materials Science and Engineering: B*, vol. 283, by A. S. Shkvarin; A. I. Merentsov; M. S. Postnikov; E. I. Patrakov; E. Betz-Guttner; L. Gregoratti; M. Amati; P. Zeller; A. N. Titov, “Morphology and composition of nanoinclusions in (Fe, Ni)_0.25_TiSe_2_“, article no. 115821, Copyright (2022), with permission from Elsevier. This content is not subject to CC BY 4.0.

The heterogeneous chemical composition of the Cr*_x_*Ti_1−_*_x_*Se_2_ system was for the first time observed and described in [12]. The observed heterogeneity led to the splitting of the Se 3d core level lines. One of the components was found to correspond to Se in TiSe_2_; the second component, which had no known analogues, was attributed to structural fragments of CrSe_2_. This confirmation was obtained directly by SPEM [13]. In the cleaved surface of the Cr_0.78_Ti_0.36_Se_2_ single crystal, regions displaying differential contrast in the Se 3d line were observed (Figure 1a–c).

In the regions of image a) delineated by the red rectangle, the shape and binding energy of the Se 3d line approximated those observed in TiSe_2_ (Figure 1b). In the region defined by the blue rectangle, values exhibited correspondence with those observed in Cr_0.83_Ti_0.26_Se_2_, but not with the data for TiSe_2_. This approach enabled the demonstration that selenium atoms with different chemical bonding characteristics are spatially distinct [12], and form separate structural fragments.

The question thus arises as to why these structural fragments form a single crystal and not independent phases. An analysis of the binding energy of the core levels of Cr 3p, Ti 3p, Ti 2p, and Cr 2p_3/2_ (Figure 1d–f) in the different fragments reveals a shift of about 0.5 eV in the binding energy, indicating that the fragments are charged relative to each other. It is evident that this is the mechanism by which the individual fragments are linked into a single crystal.

The SPEM technique is fundamental for the detection of such inhomogeneities, given that the photoelectron yield depth in this instance does not exceed 12 angstroms. This is significantly smaller than the yield depth in a scanning electron microscope (≈4 µm) or transmission electron microscope (≈1000 Å).

Another advantageous aspect of the SPEM is its capacity to achieve high lateral resolution, which enables the investigation of inhomogeneities which are relatively small in lateral dimensions. The Fe_0.25_Ni_0.25_TiSe_2_ system provides an illustrative example of such a system [14]. The single-crystal growth of a mixture of Fe_0.25_TiSe_2_ and Ni_0.25_TiSe_2_ results in the formation of a (Fe,Ni)_0.25_TiSe_2_ single crystal, which is covered with faceted single-crystal inclusions of (Ni,Fe)_4_Se_5_ (Figure 1g,h). The identical orientation of the (Ni,Fe)_4_Se_5_ crystals indicates that they have undergone coherent coupling with the lattice of the main crystal. The SPEM technique enables the determination of both the composition of inclusions and the shape of the valence band (Figure 1i–k).

### Operando characterization of InP nanowire p–n junctions

Semiconductor nanowires offer unprecedented possibilities in utilizing, combining, and modifying material properties for application in electronic, photonic, energy harvesting, or quantum information devices [15–16]. Their small footprint allows for the combination of different materials with dislocation-free interfaces and to form axial or radial heterostructures of varying material, doping, or crystal phase [17–19]. Nanowire heterostructures based on III–V semiconductors are especially promising for electronic, optoelectronic, or photovoltaic devices, as they combine a direct bandgap of tunable size with high charge carrier mobility [20]. Furthermore, they can be grown on Si substrates [21–22], which enables integration with a well-established technology platform and constrains the use of high performance, but expensive III–V materials to the active device area. The flexible geometry of nanowires standing upright on their growth substrate directly leads to gate-all-around metal-oxide-semiconductor stacks [23–24], and advanced electronic device designs such as nanowire tunneling field-effect transistors [24] or one-transistor-one-resistor cells with minimal footprint [25] can be realized. Optimizing nanowire diameter and distance further enhances the strong light adsorption of III–V materials, resulting in nanowire-based solar cells of high efficiency [26].

While such nanowire-based devices are highly promising, their performance is often limited by surface properties of the III–V semiconductor material, which furthermore can vary for individual nanowires. This includes native oxides on semiconductor surfaces and their possible removal, surface passivation, and interface defects [27]. Therefore, in-depth surface characterization of individual nanowires is urgently needed. This task is difficult for many conventional surface characterization techniques due to the nanowire geometry, and it becomes even more challenging in the case of nanowire heterostructure devices, where operando characterization of a working nanowire device under applied bias is desired. SPEM is an ideal tool for this task, as it provides topographic, chemical, and electronic information at the submicrometer scale with intrinsic surface sensitivity, even under electrical operando conditions [18]. While already conventional XPS can be used to obtain detailed information about nanowire surface, interface composition, and chemical reactions averaging over thousands or millions of nanowires in ensemble measurements [28], SPEM allows to investigate individual semiconductor nanostructures [29].

**Figure 2:**
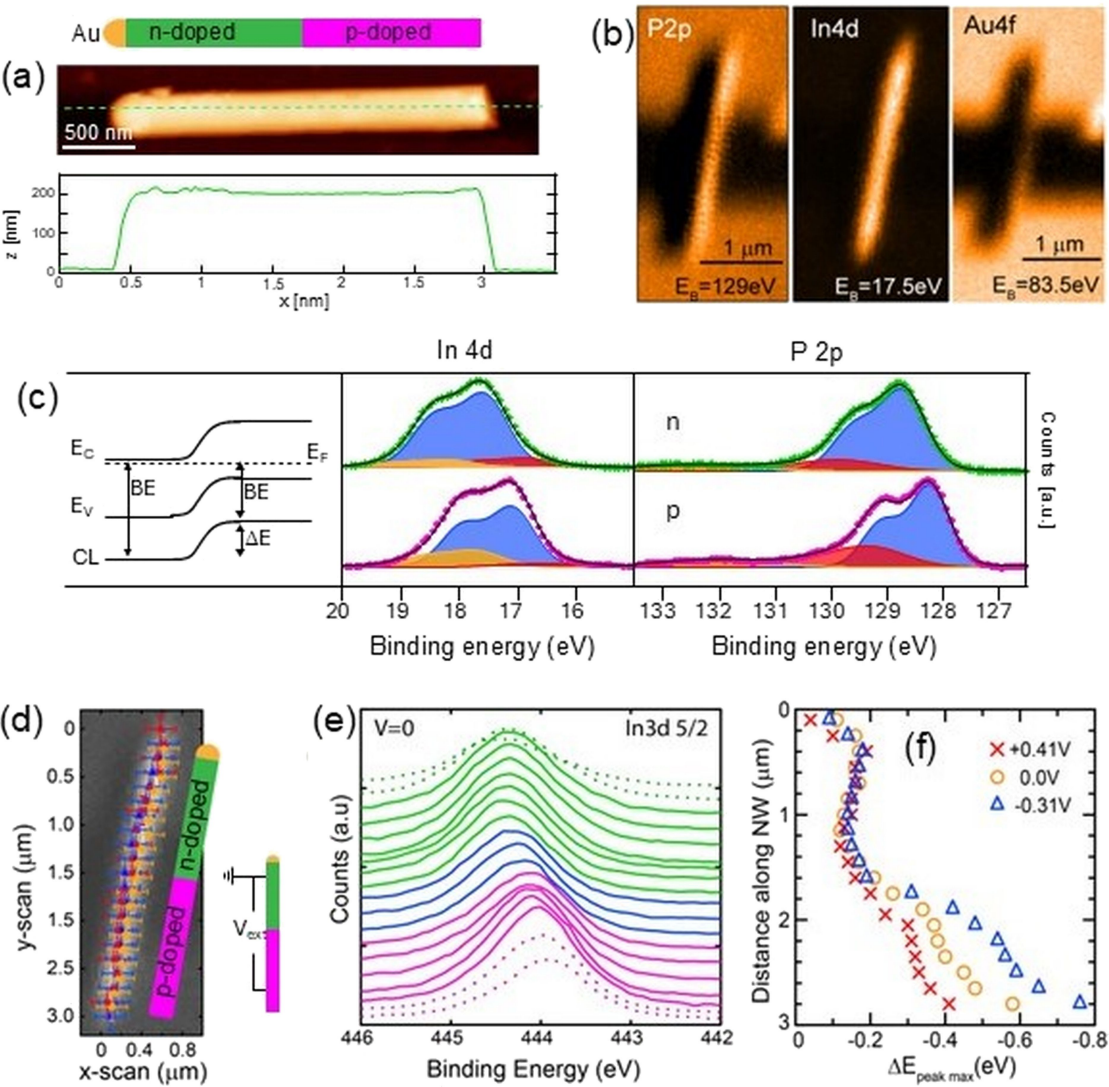
Surface characterization of InP p–n junction nanowires: a) sketched structure (top), AFM image (middle), and resulting height profile (bottom) of a nanowire with its catalytic Au particle at the top. b) SPEM images of a nanowire deposited with both ends on separately contacted Au/Ti electrodes with a trench in between, obtained around binding energies of 129 eV (P 2p, left), 17.5 eV (In 4d, middle), and 83.5 eV (Au 4f, right). c) High-resolution In 4d (middle) and P 2p (right) core-level spectra obtained at n-doped (top) and p-doped (bottom) segments of the nanowire shown in b). The band structure across the p–n junction is indicated (left). d) In 3d SPEM image of the same nanowire, positions of the spectra shown in e,f) are indicated. e) In 3d_5/2_ core-level spectra obtained along the nanowire, color-coded for n-doped segment (green), depletion region (blue), and p-doped segment (pink). f) Relative shift of fitted In 3d_5/2_ peak positions along the nanowire obtained at forward (red) and backward (blue) bias conditions and without applied bias (yellow), as indicated. Figure 2 was adapted from [18], S. R. McKibbin et al., “Operando Surface Characterization of InP Nanowire p–n Junctions”, *Nano Letters*, © 2019 American Chemical Society, distributed under the Standard ACS AuthorChoice/Editors’ Choice Usage Agreement, https://pubs.acs.org/page/policy/authorchoice_termsofuse.html. This content is not subject to CC BY 4.0.

We investigated the surface chemical composition and the surface potential across InP nanowire p–n junctions for individual nanowire devices. InP nanowires were grown on InP(111) substrates by vapor–liquid–solid growth using nanoimprint lithography for generating catalytic Au nanoparticles in a metal–organic vapor phase epitaxy (MOVPE) reactor with trimethylindium and phosphine precursors. Axial p–n junctions were created by switching from diethylzinc to hydrogen sulfide as dopant precursors halfway during nanowire growth. More growth details can be found in references [18,30]. After growth, nanowires were mechanically transferred onto suitable substrates for characterization by scanning probe microscopy and SPEM. An atomic force microscopy (AFM) image of a typical InP p–n junction nanowire is shown in Figure 2a, confirming a homogeneous shape with a nanowire length of about 2.5 µm and a diameter of about 200 nm, fluctuating only by a few nanometers along the entire nanowire.

For operando SPEM measurements, nanowires were transferred onto a prepatterned device template with flat, individually contacted Au/Ti electrodes deposited onto a SiO_2_/Si substrate, separated by a 1.5 µm wide insulating gap. Optical microscopy images of the samples including marker structures were used to directly navigate to suitable nanowires in the SPEM measurements. Figure 2b shows elemental-sensitive In 4d and P 2p SPEM images, highlighting the nanowire shape and position, which can even be noticed as a shadow in the Au 4f image, where the signal from the electrodes is attenuated. The apparently larger length of the nanowire in the In and P maps as compared to the Au map is due to the finite width of the X-ray beam, and demonstrates the convolution of nanowire shape and beam shape in the resulting image. The binding energies of the In 4d and P 2p maps were centered at 17.5 and 129 eV, respectively, and therewith below and above the binding energy of Au 4f, imaged here at 83.5 eV. This explains why inelastically scattered electrons from the Au electrodes contribute to a strong contrast in the P 2p map but cannot be seen in the In 4d map.

In addition to SPEM images, we also acquired high-resolution XP spectra from specific positions along the nanowire, as shown in Figure 2c for In 4d and P 2p core levels. A significant shift in binding energy is obvious between spectra obtained on the p-doped and on the n-doped segment of the nanowire. Since the local binding energy position of the core level directly follows the energy of the valence and conduction band and thus the band-bending at the interface between the p- and n-doped segments, the observed shift in binding energy directly reflects the in-built potential of the p–n junction at the nanowire surface. The measured binding energy shift amounts to 0.48 eV in the In 4d spectra and 0.47 eV in the P 2p spectra. Even though this is a significant and well-resolved shift, its value is surprisingly low compared with the InP band gap of 1.34 eV at room temperature. Similarly grown InP p–n junction nanowires have been reported to show an open circuit voltage between 0.6 and 0.9 eV in photovoltaic measurements [31–32]. While those open circuit voltages can be considered as the built-in potential in the nanowire bulk, the values measured by XPS result from the nanowire surface, demonstrating a weaker in-built potential at the surface. This effect can be expected from surface band bending due to defects or native oxides at the surface. This demonstrates the necessity for both bulk- and surface-sensitive measurements in order to fully understand the local potential distribution in such technologically relevant nanostructure devices.

The type and amount of surface oxide and defects can be obtained from XPS peak fitting, as presented in Figure 2c, assuming a Shirley background and Voigt doublet components (more fitting components can be found in [18]). Both the In 4d and P 2p spectra were fitted by a dominating component, attributed to the InP bulk signal, and two smaller doublets each. For P 2p, these smaller components have a 0.95 and a 3.5 eV higher binding energy than that of the bulk component, respectively. According to the literature, the high binding energy component can be attributed to P in a +5 oxidation state, as in InPO_4_, and the other one as P^0^, probably due to metallic P defects at the nanowire surface [18,33–34]. A small oxide component is also found in the In 4d spectra, at a binding energy which is 0.6 eV higher than that of the bulk component. In addition, a small In 4d component with 0.75 lower binding energy as compared to that of the bulk peak is also observed. This component might be due to metallic In or to In alloying with Au, either from the catalytic Au particle at the top of the nanowire, or from the Au electrodes.

After analyzing the chemical composition and electrical potential distribution of the nanowire surface across the p–n junction in the unbiased case, we were ready to proceed to operando measurements during forward and backward applied bias. These can be realized by biasing the two electrodes on which the nanowire is resting with its p- and n-doped ends. In practice, the electrode with the n-doped segment was grounded, while a bias of +0.4, 0, or −0.3 V was applied to the electrode with the p-doped segment, resulting in forward-biased, unbiased, and backward-biased conditions of the p–n junction. High-resolution In 3d core-level spectra were obtained along the nanowire with a step width of about 130 nm, roughly equaling the size of the X-ray beam, as indicated in Figure 2d. The resulting In 3d_5/2_ spectra are shown in Figure 2e for the unbiased case, highlighting the p-doped and n-doped nanowire segments with the depletion zone in between, while the energy shift of fitted peak positions along the nanowire is plotted in Figure 2f for both unbiased, forward biased, and backward biased conditions. The energy shift between p- and n-doped segments decreases to about 0.2 eV upon forward bias and increases to about 0.6 eV upon backward bias. While most of the bias drops across the depletion region, as expected for a sharp p–n junction, an additional voltage drop is observed along the p-doped segment.

Our results provide an important step in understanding the electrical behavior of a nanowire p–n junction under device operation with high spatial resolution. We correlate that to the chemical surface composition, distinguishing between surface and bulk effects on the path towards improved surface properties of nanowire solar cells with further enhanced efficiency. Furthermore, they demonstrate the large suitability of SPEM for operando studies of technologically relevant nanoscale heterostructure devices.

### Self-organized NiO microcavity arrays fabricated by thermal treatments

In the class of the very few p-type oxides, NiO stands out as one of the most versatile and promising materials in diverse applications including supercapacitors [35–36] fast spintronics [37], electrochromic devices [38–39], catalysts, and gas sensors [40–41], [42–43]. The characteristic p-type conductivity of this transition metal oxide is related to the presence of oxygen interstitials and the inherent nickel deficiency which leads to the formation Ni^3+^ to reach charge neutrality. Additionally, NiO exhibits a wide bandgap, which also prompts considerable research interest. The properties of NiO are highly dependent on the synthesis method, owing to the variable dimensionality, morphology, crystalline orientation, and defect structure [44]. Therefore, it is important to increase research efforts to improve the knowledge and understanding of the properties of this multifaceted material in order to better predict and tailor its role in diverse applications.

XPS has been extensively employed in the study of NiO, leading to relevant insights regarding its electronic structure, where emphasis is common for the study of the Ni 2p signal up to now. However, some discrepancies are still reported on the correct assignment of XPS features [45]. Additionally, less research has been done so far in the analysis of the Ni 3p signal, the study of which can be occasionally necessary in order to avoid overlapping of Ni 2p signal with other XPS contributions in doped NiO or Ni-based compounds.

**Figure 3:**
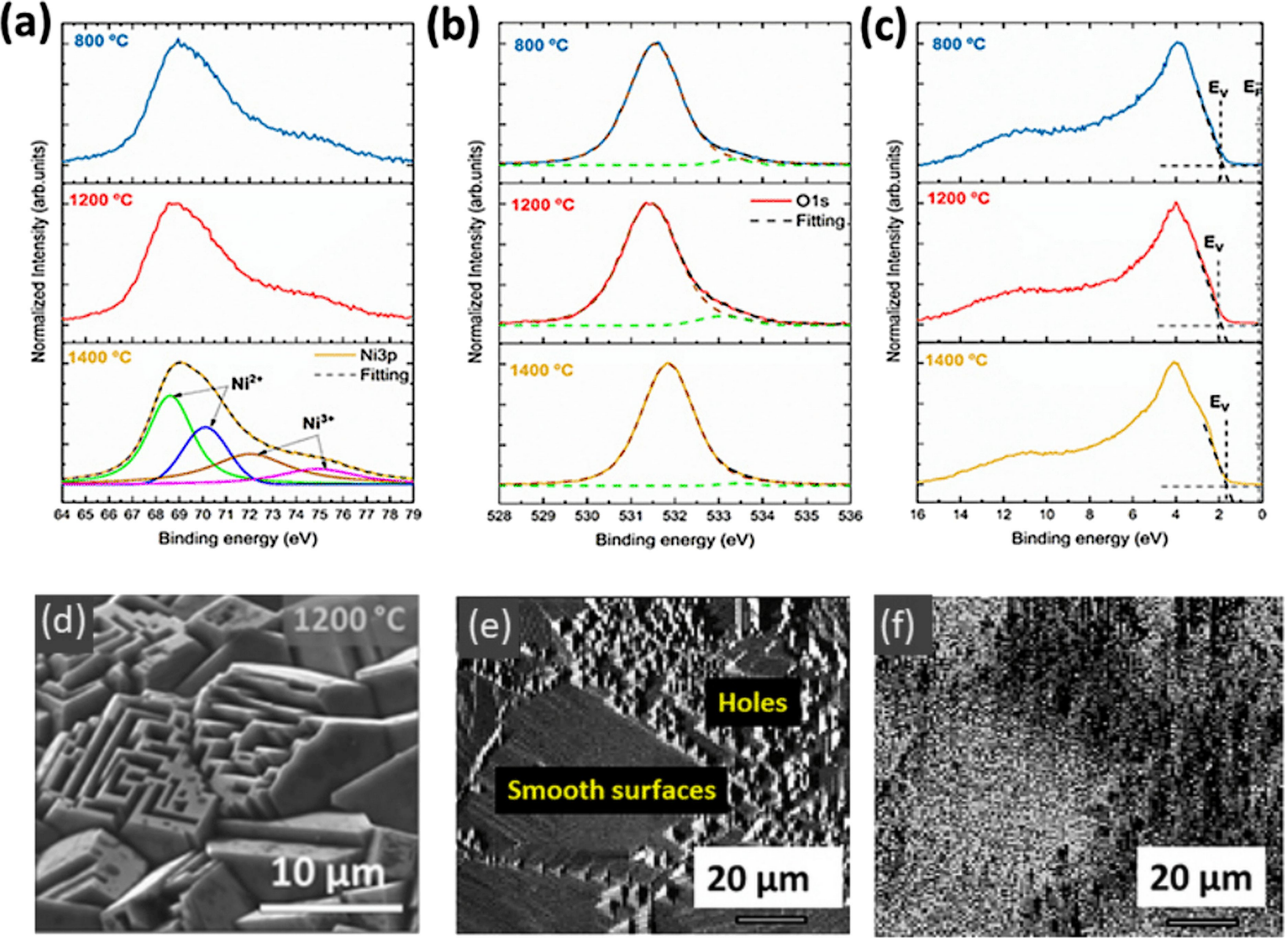
XPS spectra from a) Ni 3p, b) O 1s core levels, and c) valence band region from NiO samples sintered at 800, 1200, and 1400 °C. d) SEM image and e) XPS image acquired with the Ni 3p signal, from the sample treated at 1200 °C. f) XPS energy filtered image from e) corresponding to the Ni^2+^/Ni^3+^ ratio. Adapted with permission from *Cryst. Growth Des.* 2020, 20, 4082–4091. Copyright 2020 American Chemical Society. Figure 3 was reprinted with permission from [46], Copyright 2020 American Chemical Society. This content is not subject to CC BY 4.0.

Taeño et al. [46] recently reported the study of NiO microcrystals with self-organized microcavities synthesized by a vapor–solid process at temperatures ranging from 800 to 1500 °C under a controlled Ar atmosphere. In that study, XPS analysis was performed in combination with complementary microscopy and spectroscopy techniques. The analyzed NiO microstructures exhibit dimensions in the micrometric range, with preferential {100} surface texturing, where self-ordered cavities were formed at temperatures between 1000–1200 °C. The lateral surfaces of those small cavities or pinholes correspond to the {111} family of planes, which tend to be more reactive owing to the higher concentration of Ni^3+^. Diverse phenomena, including Ni diffusion and inhomogeneous strain, were considered to occur during the growth process, leading to NiO samples with variable properties. Raman spectroscopy indicated changes between the relative intensity of first order modes and the 2M mode due to the variable lattice disorder induced in the samples during the growth process. Cathodoluminescence measurements demonstrated the presence of a visible emission at 2.5 eV associated with Ni deficiency, whose relative intensity varies as a function of the thermal treatment and the consequent structure of defects. The NiO samples showed additional luminescence due to Ni interstitial and d–d transitions. In that work, XPS measurements carried out in the ESCA microscopy beamline at Elettra Synchrotron facilities contributed with significant knowledge to the study of the surface properties of the NiO samples. Particularly, advantage was taken from the SPEM which can work both in spectroscopy and imaging modes. Figure 3 shows Ni 3p, O 1s, and valence band XPS spectra from samples annealed at diverse temperatures, which exhibit variable morphological, crystallographic, and optical properties. Regarding the Ni 3p signal, less-explored so far, four main bands were observed after signal deconvolution (Figure 3a). Bands at lower binding energy, 68.6 and 70.1 eV, were related to Ni^2+^ in the NiO lattice, while doublet at 72.0 and 74.9 eV was associated with Ni^3+^. An increase in the Ni^3+^/Ni^2+^ ratio was observed for the samples treated at higher temperature. The O 1s core levels from all the probed samples were dominated by a band at 531.5 eV related to O^2−^ in NiO, while a lower intensity band was observed at 533.2 eV associated with adsorbed oxygen species (Figure 3b). Preferential oxygen absorption is expected for {111} surfaces which were observed in the cavities formed at the surface of some samples. Regarding the valence band photoelectron spectra, mainly formed by O 2p and Ni 3d contributions, a higher p-type character was observed for the samples annealed at 1400 °C, based on the position of the Fermi level relative to the VB maximum (Figure 3c). Special attention was paid to the study of the sample grown at 1200 °C with a high concentration of cavities (Figure 3d). XPS image acquired with the Ni 3p signal (Figure 3e) and the corresponding energy-filtered image (Figure 3f) indicated variable Ni^3+^/Ni^2+^ ratio at the surface of the probed sample, where either flat regions or areas with cavities were formed. In particular, a higher concentration of Ni^3+^ observed in the regions with surface cavities is also associated to a lower presence of absorbed oxygen. This can be correlated with surface defects responsible for the increase in the relative intensity of the near-infrared emission observed in the corresponding CL spectrum.

XPS measurements provide valuable information on the electronic structure and properties of the NiO samples, which strongly support the development of potential applications including gas sensors and optical resonators [43,47].

## Conclusion

SPEM is a synchrotron-based technique combining XPS, submicron spatial resolution, and chemical imaging capabilities. The instrument hosted at the Elettra synchrotron research center, in operation since 1993, has been recently upgraded to fulfil the increasingly demanding needs of in situ and operando experiments. The examples of materials characterization shown in the review demonstrate its usefulness in the investigation on nanostructured materials regardless of their morphology. This feature is of particular interest when device prototypes, such as sensors and batteries, must be analyzed in real conditions. The combination of imaging and spectroscopy can provide an elemental, chemical, and electronic mapping of sample surfaces in the pristine form or after/during their response to external stimuli such as temperature changes, electric and magnetic fields, and light and gas exposure.

In the first example chemical heterogeneous layered transition metal dichalcogenides were analyzed showing the importance of spatial resolution in their characterization. In the second example, the electronic behavior of InP nanowire p–n junctions heterostructures has been investigated under applied bias correlating it to the chemical surface composition and surface/bulk effects. In the last example, the results of a SPEM investigation of self-organized NiO microcavity arrays exhibiting dimensions in the micrometric range concludes this overview of nanostructured materials characterized by SPEM.

After describing the current state of the art of SPEM capabilities in the characterization of nanostructured materials, it is worth spending a few sentences about the future of this technique in the light of the very fast evolution of synchrotron sources towards diffraction-limited performance. Two main parameters that characterize the SPEM technique are its energy and spatial resolution. The first one, which defines the chemical sensitivity of the instrument, is notably higher than what is generally available at spectroscopy stations. The new generations of light sources will dramatically improve the optical transmission of X-rays allowing more photons at the samples. Those may be spent to increase energy resolutions at values lower than 80–100 meV and also at higher energies. Similar improvements may also be reached in the spatial resolution; however, any increase in photon density goes with an enhancement of undesired beam-induced effects, such as surface chemical changes, local charging, and space-charge effects. To partially mitigate this effects, strong efforts must be spent in the development of new photoelectron detection systems capable of collecting the majority of electrons, currently wasted due to the limited acceptance angles of hemispherical analyzers, while guaranteeing adequate energy resolutions.

## Experimental

**Figure 4:**
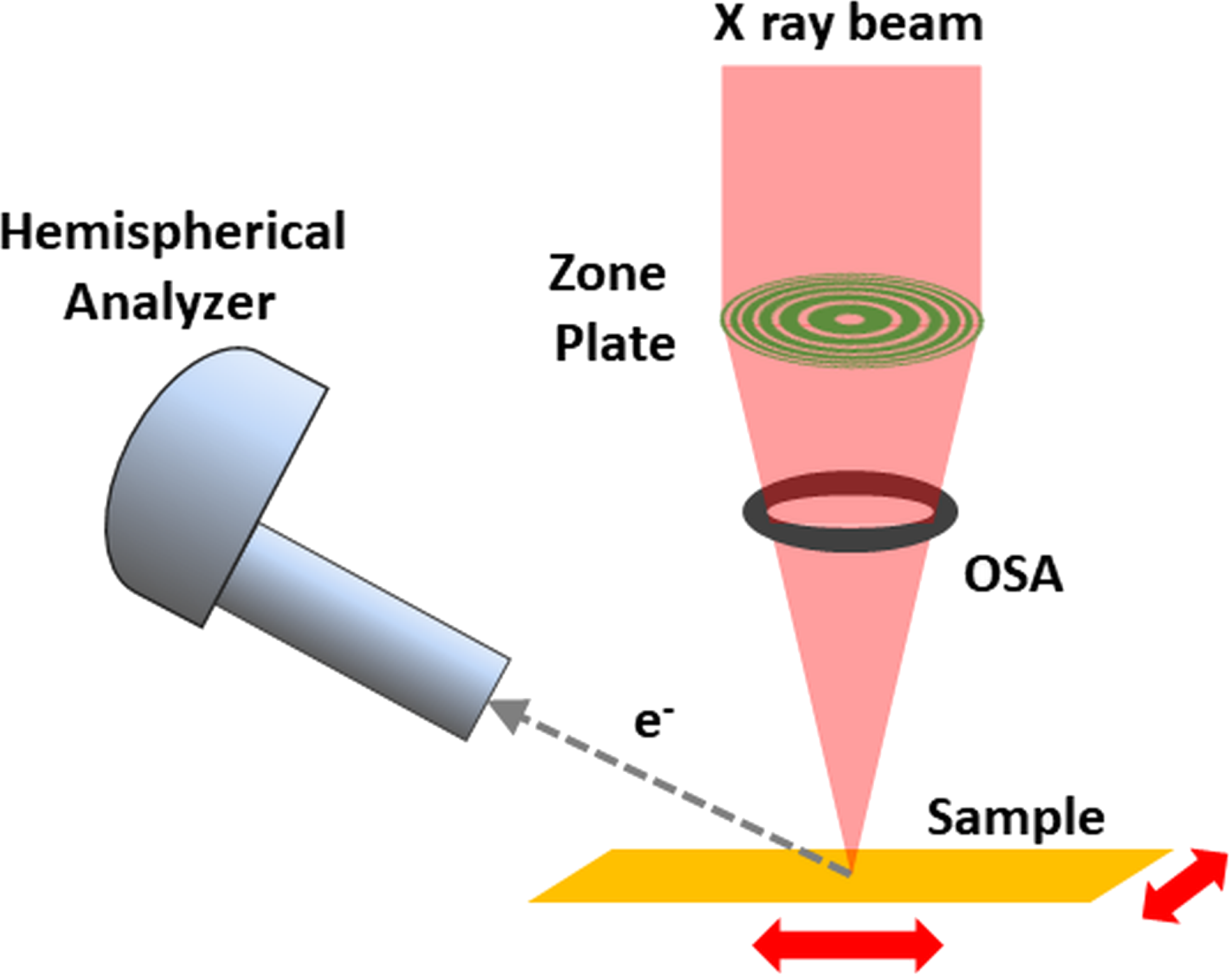
Geometrical displacement of the SPEM focusing optical elements, sample, and electron analyzer.

The SPEM hosted at the ESCA microscopy beamline at Elettra uses diffraction optics to focus the incoming X-ray beam shaped by the photon transport optics to a 130 nm Gaussian-shaped spot. As shown with a sketch in Figure 4, focusing optics are composed by a zone plate (ZP) and an order sorting aperture which selects one of the diffracted spots generated by the ZP. The focal distance depends on the geometrical parameters of the ZP and on the photon energy. At the experimental conditions of the experiments reported in this article, the focal distance was ≈10 mm. The narrow X-ray spot normally impinges the sample surface generating a cloud of photoelectrons, part of which are collected by a hemispherical electron analyzer pointing at the sample at a 30° take off angle. The choice of such angle is strongly constrained by the crowded area around the sample where the sample and optics holders and analyzer nose must be located within a space of a few millimeters. The further displacement of the electron analyzer increases the natural surface sensitivity of XPS; depending on the kinetic energy of the detected photoelectrons, the probing depth typically stays in the range of 3–10 monolayers. It is straightforward to understand that such feature may uniquely enhance the results of some characterization parameters, but may represent a limit for others.

As shown in Figure 4, the SPEM at Elettra uses a fixed incoming focused X-ray beam, (i.e., the sample is raster scanned with respect to it to create chemical maps). At each step/pixel of the scan/image the number of photoelectrons with a specific kinetic energy corresponding to a particular atomic element chemical state is recorded. The contrast in the final map is then showing the spatial concentration of such state in the analyzed area.

At the photon energy used for the experiments described above (650 eV), the SPEM system guarantees an overall energy resolution in the range of 200–350 meV.

## Data Availability

Data sharing is not applicable as no new data was generated or analyzed in this study.
